# Study on the Correlation Between Microbial Community Composition and Flavor of Traditional Ordos Air-Dried Meat

**DOI:** 10.3390/foods15091510

**Published:** 2026-04-27

**Authors:** Jiaqi Zhang, Lina Sun, Erke Sun, Shiqi Hao, Shuo Li, Ru Yan, Ye Jin, Lihua Zhao, Xueying Sun

**Affiliations:** 1 College of Food Science and Engineering, Inner Mongolia Agricultural University, Hohhot 010018, China; ybdgh957@163.com (J.Z.); beautysunlina@126.com (L.S.); sun15848107303@163.com (E.S.); haosq_imau@163.com (S.H.); lishuo1245431719@163.com (S.L.); yanr2021@163.com (R.Y.); jinyeyc@sohu.com (Y.J.); 2 Beef and Mutton Quality Identification and Safe Processing, Inner Mongolia Autonomous Region Engineering Research Center, Hohhot 010018, China

**Keywords:** Ordos air-dried meat, microbial community composition, meat quality, flavor compounds

## Abstract

Traditional air-dried meat from Ordos, Inner Mongolia, is a distinctive fermented meat product with unique ethnic and regional features. However, systematic studies on its quality and flavor variations across production regions remain limited. This study characterized 15 traditional Ordos air-dried meat samples (3 biological replicates per region) from five representative areas (Dalad Banner, Otog Banner, Otog Front Banner, Wushen Banner, Ejin Horo Banner) via physicochemical analysis, high-throughput 16S rRNA gene sequencing, and GC-MS. Results showed pH 5.63–5.73 and water activity (Aw) 0.61–0.66. Wushen Banner samples had significantly lower TVB-N and TBARS contents. Proteobacteria and Firmicutes dominated, with marked regional divergence at the genus level. Ninety-one volatile compounds were identified, 11 as key odor-active components. This study clarifies regional quality discrepancies and interrelationships among microbiota, flavor, and physicochemical traits, providing a theoretical basis for process optimization and industrial development.

## 1. Introduction

Ordos traditional air-dried meat is produced using conventional techniques, with natural air-drying carried out under low-temperature conditions from December to April each year, typically at −10 to 10 °C and relative humidity. Characterized by its compact size, rich nutritional profile, and unique flavor, it has become a product with a distinct ethnic and traditional identity. Production is primarily concentrated in Inner Mongolia, Xinjiang, Qinghai, and Tibet [1]. In recent years, growing public interest in traditional dietary culture has driven the growth in market demand for air-dried meat products, the market demand for high-quality traditional air-dried meat has increased year by year. Unlike industrialized fermented meat products, the quality formation and flavor development of traditional naturally air-dried meat are completely driven by the natural microbial community from raw materials and processing environment, and the composition structure and metabolic activity of microorganisms are the core factors determining the final product quality, flavor and edible safety [2]. Li et al. used this technology to analyze the dynamic changes of bacterial community during the processing of dry-cured duck, and found that the relative abundance of dominant genera such as Staphylococcus, Vibrio and Lactococcus gradually increased with the extension of air-drying time [3]. Min et al. analyzed the microbial community composition of chilled chicken stored at 4 °C under aerobic conditions, and the results showed that Pseudomonas, Acinetobacter, Aeromonas, Shewanella were the predominant spoilage bacteria in chicken thighs, Myroides, Yersinia and Shewanella were the newly reported important contributors to the spoilage-related microflora [4].

Microorganisms of diverse species and abundance participate in the generation, transformation, and accumulation of flavor compounds through their unique metabolic activities during the processing and storage of air-dried meat [5]. By combining high-throughput sequencing with metabolomics, Qin et al. analyzed the correlation between microbial communities and metabolites in four types of Yunnan air-dried hams [6]. The study found that the dominant genera including *Halomonas* and *Staphylococcus* can significantly promote the generation of aldehydes and esters through metabolic pathways such as fatty acid β-oxidation and amino acid deamination, which directly confirmed the significant correlation between microbial metabolic activities and the formation of key flavor compounds in meat products [7,8].

The traditional air-dried meat production process is simple but time-consuming, and has several drawbacks such as complex procedures, basic equipment, and low mechanization level [9]. During long-term air-drying and storage, the lack of effective protective measures often results in products with quality defects including coarse texture, dull color, and single flavor profile. In addition, harmful substances generated during this period shorten shelf life, reduce edible quality, and fail to meet diverse consumer demands while also posing potential health risks to consumers [10]. However, most existing studies have focused on the overall quality characteristics or microbial changes during processing of air-dried meat from other regions, while few have systematically explored the regional differences in microbial community composition and flavor formation mechanisms of traditional air-dried meat from Ordos [11]. Moreover, the specific effects of local climatic conditions and traditional processing practices on microbial succession and flavor development in Ordos air-dried meat remain unclear [12]. The lack of such basic research limits the scientific understanding of its quality formation mechanism and hinders the standardization and industrialization of production [13].

Although there have been a lot of studies on the quality and flavor formation mechanism of traditional air-dried meat, there is still a lack of systematic research on the key factors affecting the microbial community composition and flavor quality of traditional air-dried meat from different producing areas of Ordos. Therefore, it is urgent to systematically analyze the microbial community composition and core dominant genera of traditional air-dried meat from different regions of Ordos, and clarify the correlation between dominant microorganisms and flavor substances, so as to provide a scientific theoretical basis for the standardized production of this traditional product. This study aims to clarify the differences in quality characteristics and microbial community composition of traditional air-dried meat from different regions of Ordos, screen its key aroma-active compounds via ROAV calculation, and reveal the correlation between dominant microbial genera, physicochemical quality and key flavor compounds. The results of this study are expected to lay a theoretical foundation for the standardized production and quality regulation of traditional Ordos air-dried meat, and provide scientific support for the inheritance of ethnic food culture and the high-quality development of local characteristic meat industry.

## 2. Materials and Methods

### 2.1. Experimental Materials

Traditional naturally air-dried meat samples were collected from five representative administrative regions in Ordos City, Inner Mongolia, China, including Dalad Banner, Otog Banner, Otog Front Banner, Wushen Banner, and Ejin Horo Banner. Three parallel final product samples were collected from a local traditional processor in each region, and all samples were from the same production batch to ensure parallelism, Samples with mildew, rancid odor, or inconsistent production cycle were excluded from the study. All samples were manufactured following local traditional handcrafted processes. Fresh mutton from sheep raised on local grasslands was used as the raw material, and the entire process of natural fermentation and air-drying was completed in a well-ventilated, shaded indoor natural environment between December 2023 and April 2024, without any artificial inoculation of starter cultures or temperature and humidity control. All samples were the final commercial products of the core production batch in the season, which can represent the typical quality and flavor characteristics of traditional air-dried meat in the corresponding producing area.

Based on their origin, the samples were assigned to the following groups: Dalad Banner (DC), Otog Banner (EHC), Otog Front Banner (EC), Wushen Banner (WC), and Ejin Horo Banner (YC). After sampling, all samples were immediately placed into sterile sealed sampling bags and stored at 4 °C with ice packs to maintain a stable low temperature. The samples were transported to the laboratory within 24 h. During transportation, the samples were protected from direct sunlight, severe vibration and temperature fluctuations, and the integrity of the sterile packaging was strictly inspected prior to subsequent laboratory treatment. Each sample was divided into two portions: one was stored at −80 °C ultra-low temperature freezer for subsequent microbial high-throughput sequencing analysis, and the other was vacuum packaged and stored at 4 °C refrigerator for physicochemical indexes, sensory evaluation and volatile flavor compounds determination.

### 2.2. Test Reagents

Trichloroacetic acid, sodium chloride, magnesium oxide, boric acid, thiobarbituric acid, n-hexane, chloroform, anhydrous sodium sulfate, sodium hydroxide, sulfuric acid, anhydrous ethanol and sodium bicarbonate were all of analytical grade, purchased from Sinopharm Chemical Reagent Co., Ltd., Shanghai, China; methanol and acetonitrile were of chromatographic grade, purchased from Thermo Fisher Scientific Inc., Waltham, MA, USA; acetone was of analytical grade, purchased from Tianjin Fengchuan Chemical Reagent Technology Co., Ltd., Tianjin, China. All solutions used in the test were prepared in accordance with national standard (GB) methods and literature requirements.

### 2.3. Instruments and Equipment

HD-3A intelligent water activity meter (Wuxi Huake Instrument and Meter Co., Ltd., Wuxi, China); AL204 1/10000 electronic analytical balance, PB-STAR carcass pH direct meter (Mettler-Toledo Instruments Co., Ltd., Shanghai, China); TCP2 automatic color difference meter (Beijing Aoyikemi Optoelectronics Co., Ltd., Shanghai, China); Trace 1300 ISQ gas chromatography–mass spectrometry (GC-MS) system, 1260 high performance liquid chromatography (HPLC) system (Thermo Fisher Scientific, Waltham, MA, USA); HH-2 digital display constant temperature water bath (Jintan Jiangnan Instrument Factory, Jintan, China); LHS-10CL constant temperature and humidity chamber (Shanghai Yiheng Technology Co., Ltd., Shanghai, China).

### 2.4. Determination of Physicochemical Indicators

#### 2.4.1. pH Measurement

The pH values of the samples were measured following the protocol described by Yao et al. (2019) [14] with minor modifications. 2 g of the sample were weighed and thoroughly mixed with 18 mL of physiological saline. The mixture was then stirred with a magnetic stirrer for 30 min, and pH of the resulting solution was measured. Triplicate to quintuplicate parallel analyses were conducted on each group of samples.

#### 2.4.2. A_w_ Measurement

The A_w_ of the samples was determined following the protocol described by Aheto et al. (2019) [15] with minor modifications. 10 g of the sample were chopped and placed into a measurement cup, and water activity was measured using an A_w_ meter (Wuxi Huake Instrument and Meter Co., Ltd., Wuxi, China). The measurement was repeated thrice per group, and the average value was calculated. Triplicate to quintuplicate parallel analyses were conducted on each group of samples.

#### 2.4.3. Determination of TVB-N

The TVB-N content of the samples was determined following the protocol described by Li et al. (2018) [16] with minor modifications. 10 g of the homogenized sample was accurately weighed into a 150 mL conical flask, 100 mL of distilled water was added, and the mixture was oscillated for 30 min and then stood for 10 min. 25 mL of the supernatant was absorbed into the digestion tube of the Kjeldahl nitrogen analyzer (Shanghai Peiou Analytical Instrument Co., Ltd., Shanghai, China), and 1 g of magnesium oxide (MgO) was added. The instrument parameters were set as follows: the volume of boric acid absorption solution was 30 mL, and the distillation time was 180 s. Each sample was determined in triplicate, and the results were expressed in g/kg of sample.

#### 2.4.4. Determination of TBARs

The TBARs content of the samples was determined following the protocol described by Grotta et al. (2017) [17] with minor modifications. 5 g of the sample was accurately weighed, mixed with 25 mL of 7.5% trichloroacetic acid (TCA) solution (containing 0.1% EDTA), fully stirred for 30 min, and then filtered with double-layer filter paper. 5 mL of the filtrate was mixed with 5 mL of 0.02 mol/L thiobarbituric acid (TBA) solution, heated in a boiling water bath for 40 min, and then cooled to room temperature with running water. 5 mL of chloroform was added to the mixture, which was fully oscillated and stood for layering. The absorbance of the upper aqueous phase was measured at 532 nm and 600 nm, with the TCA solution as the blank control. Each sample was determined in triplicate, and the results were expressed in g/kg of sample.

### 2.5. Sensory Evaluation

The Sensory evaluation of the samples was determined following the protocol described by Wang et al. (2023) [18] with minor modifications. Ten panelists with equal gender distribution and experience in meat product sensory evaluation were recruited from the Department of Food Science. Sensory evaluation was conducted in dedicated, interference-free sensory booths at room temperature, illuminated by standard cool white fluorescent lighting with an intensity of 500–600 lx. Meat samples were trimmed to remove fascia, cut into uniform 2 mm thick slices, and divided into approximately 10 g portions. All samples were coded with random three-digit numbers and presented in random order. Panelists independently scored color, aroma, tissue structure, and texture of samples using the structured scoring scale detailed in Table 1. Each sample was scored within 5 min, with a 5 min interval between samples. Panelists cleansed their palates and neutralized residual taste with unsalted soda crackers and mineral water between evaluations. The entire evaluation was completed within 2 h.

### 2.6. Determination of Microbial Community Composition

#### 2.6.1. DNA Extraction

Total microbial genome DNA was extracted from the meat samples using E.Z.N.A.^®^ Soil DNA Kit (Omega Bio-tek, Norcross, GA, USA) strictly according to the manufacturer’s operating instructions [19]. The integrity of the extracted DNA was detected by 1% agarose gel electrophoresis, and the concentration and purity of DNA were determined by NanoDrop 2000 ultra-micro spectrophotometer (Thermo Fisher Scientific, Waltham, MA, USA). The qualified DNA samples were stored at −20 °C for subsequent PCR amplification.

#### 2.6.2. PCR Amplification and Sequencing Library Construction

PCR amplification was performed using extracted DNA as the template with barcode-labeled primers. The full-length 16S rRNA gene was amplified with primers 27F (5′-AGRGTTYGATYMTGGCTCAG-3′) and 1492R (5′-RGYTACCTTGTTACGACTT-3′) [20], whereas the internal transcribed spacer (ITS) region was targeted using primers ITS1F and ITS4R [21].

Each 20 μL PCR mixture consisted of 4 μL 5× FastPfu Buffer, 2 μL 2.5 mM dNTPs, 0.8 μL 5 μM forward primer, 0.8 μL 5 μM reverse primer, 0.4 μL FastPfu DNA Polymerase, 0.2 μL bovine serum albumin (BSA), 10 ng template DNA, and sterile double-distilled water.

Triplicate reactions were set for each sample. Thermal cycling conditions included an initial denaturation at 95 °C for 3 min, 27 cycles of 95 °C for 30 s, 60 °C for 30 s and 72 °C for 30 s, followed by a final extension at 72 °C for 10 min, with amplicons stored at 4 °C. PCR products were detected by 2% agarose gel electrophoresis, purified with magnetic beads, quantified via a Qubit 4.0 Fluorometer, and pooled at ratios corresponding to the required sequencing depth. Library preparation was completed using the SMRTbell^®^ Prep Kit 3.0 (Pacific Biosciences, Menlo Park, CA, USA) through DNA damage repair, end repair and adapter ligation. Sequencing was conducted on the Pacbio Sequel IIe System by Shanghai Majorbio Bio-pharm Technology Co., Ltd. (Shanghai, China). High-Fidelity (HiFi) reads were generated from raw subreads by circular consensus sequencing (CCS) with SMRT Link v11.0 for subsequent data analysis.

### 2.7. Determination of Volatile Flavor Compounds

#### Method for Determining Volatile Flavor Compounds

According to the method of Liu et al. [22] with slight modifications. The volatile flavor compounds of the samples were extracted by headspace solid-phase microextraction (HS-SPME) method. Briefly, 5 g of minced sample was accurately weighed into a 20 mL headspace vial, which was immediately sealed with a PTFE-silicone septum. The sample was balanced in a 60 °C constant temperature water bath for 10 min, and A pre-conditioned solid phase microextraction (SPME) fiber was then inserted into the headspace, and adsorbed at 60 °C for 40 min under continuous stirring. After adsorption, the fiber was immediately transferred to the injection port of the gas chromatograph, and desorbed at 250 °C for 3 min.

GC conditions: Volatile compounds were separated on capillary column (30 m × 0.25 mm × 0.25 μm). High-purity helium (purity ≥ 99.99%) was employed as the carrier gas at a constant flow rate of 1.0 mL/min. The injector temperature was fixed at 250 °C, and splitless injection was conducted with a splitless time of 1 min. The oven temperature program was set as: initial temperature held at 40 °C for 3 min; ramped to 150 °C at 4 °C/min and maintained for 1 min; further increased to 200 °C at 5 °C/min; and finally raised to 230 °C at 20 °C/min and held for 5 min.

MS conditions: The ion source temperature was maintained at 250 °C, and electron ionization (EI) mode was adopted with an electron energy of 70 eV. The mass scanning range was set at 30–400 m/z, and the solvent delay time was set to 1.0 min. Volatile compounds were identified by matching the acquired mass spectra with the NIST 20, Wiley and meanlib databases. A match factor ≥ 800 was used as the criterion for positive identification. The results were expressed as the percentage of each compound relative to the total identified compounds.

The relative contribution of each aroma component to the overall flavor of the sample is commonly expressed as the relative odor activity value (ROAV), which is calculated using the following formula:

(1) ROAV = 100 × CA/CMAX × TMAX/TA

where CA = relative percentage content of a specific volatile flavor compound A; CMAX = relative percentage content of the volatile flavor compound with the highest contribution to the overall flavor of the sample; TMAX = odor threshold of the volatile flavor compound with the highest contribution to the overall flavor of the sample; TA = the odor threshold of a specific volatile flavor compound A. All samples were subjected to identical sample pretreatment and instrumental analysis conditions to minimize matrix effect arising from the heterogeneity of traditional air-dried mutton samples, which ensured the accuracy of the relative quantitation of volatile flavor compounds and the subsequent calculation of ROAV.

### 2.8. Statistical Analyses

The bioinformatics analysis of microbial community was completed on the Majorbio Cloud Platform [23]. Mothur software (Version 1.48.0) was used for the calculation of alpha diversity indexes of microbial community [24], and the Kruskal–Wallis H non-parametric test was used for inter-group difference analysis of alpha diversity indexes due to the non-normal distribution of the data. LEfSe analysis (LDA score > 2, *p* < 0.05) was used to screen the microbial taxa with significant differences in abundance among different sample groups [25]. Statistical analysis of physicochemical indexes, sensory evaluation and volatile flavor compounds was performed using SPSS 26.0 software. Prior to parametric statistical analysis, the Shapiro–Wilk test and Levene’s test were conducted to examine the normality of data distribution and homogeneity of variances, respectively. Data conforming to normal distribution and homogeneous variances were analyzed by one-way analysis of variance (ANOVA), followed by Duncan’s multiple range test for pairwise comparisons between groups. For non-normally distributed data, the Kruskal–Wallis H non-parametric test was used for inter-group comparison. A significance level of *p* < 0.05 was adopted for all statistical analyses, and results were expressed as mean ± standard deviation (SD). Pearson’s correlation analysis was used to analyze the correlations among dominant bacterial genera (relative abundance > 1%), physicochemical indexes and key flavor compounds, and the corresponding correlation network diagrams were constructed. All data visualization was performed using Origin 2018 software.

### 2.9. Data Availability Statement

The raw 16S rRNA gene sequencing data generated in this study have been deposited in the NCBI Sequence Read Archive (SRA) under the BioProject accession number PRJNA1432322 (Temporary Submission ID: SUB16040136). The data are scheduled for public release on 7 March 2026 and will be accessible at: https://www.ncbi.nlm.nih.gov/sra/PRJNA1432322 (accessed on 22 April 2026).

## 3. Results

### 3.1. Determination of Physicochemical Quality Indicators of Ordos Traditional Air-Dried Meat

**Figure 1:**
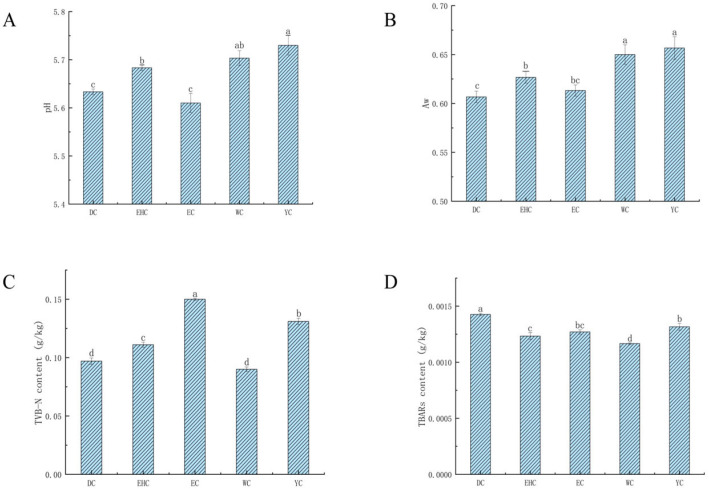
Physicochemical quality characteristics of Traditional Air-Dried Meat from Different Regions of Ordos. (**A**) pH Values of Traditional Air-Dried Meat from Different Regions in Ordos. (**B**) Aw of Traditional Air-Dried Meat from Different Regions of Ordos. (**C**) TVB-N of Traditional Air-Dried Meat from Different Regions of Ordos. (**D**) TBARs of Traditional Air-Dried Meat from Different Regions of Ordos. Different lowercase letters above the bars indicate significant differences (*p* < 0.05) based on one-way ANOVA followed by Duncan’s multiple range test. Data are expressed as mean ± standard deviation (SD). Abbreviations: EC, Otog Front Banner; DC, Dalad Banner; YC, Ejin Horo Banner; WC, Wushen Banner; EHC, Otog Banner.

pH value is one of the key quality indicators for meat products. Raw meat with a pH above 6 exhibits increased microbial risks and poor water-holding capacity, whereas a pH below 5.5 imparts a sour taste that impairs the overall sensory properties. Air-dried meat with a pH ranging from 5.5 to 6.0 is more likely to achieve desirable texture and color [26]. As shown in Figure 1A, the pH values of traditional air-dried meat samples from different regions of Ordos varied, with the mean pH values ranging from 5.63 to 5.73. Among the samples, the EC group exhibited the lowest pH (*p* < 0.05), whereas the YC group showed the highest pH (*p* < 0.05). The pH differences among regions are mainly caused by the differential metabolism of dominant microorganisms, which produce organic acids and regulate the acid-base environment of the meat matrix [27].

A relatively low A_w_ can effectively inhibit the growth of spoilage microorganisms [28]. As shown in Figure 1B, the DC group exhibited the lowest A_w_, whereas the YC group had the highest. The mean A_w_ of traditional air-dried meat samples from different regions of Ordos ranged from 0.61 to 0.66. The low A_w_ level of Ordos air-dried meat is notably lower than industrial processed meat products [29]. This feature is formed by the cold-dry climate of Ordos and traditional air-drying crafts, which endows the product with better storage stability. Currently, some countries have established regulatory standards for A_w_ as part of food safety control. Specifically, the United States Food Inspection Service stipulates that the A_w_ value of dried meat products remain below 0.70 when directly exposed to air [30]. All the tested traditional air-dried meat samples from Ordos complied with this standard.

TVB-N is widely used as an indicator to evaluate the freshness of meat products [31]. High TVB-N values negatively impact product quality, safety, nutritional value, and flavor. As shown in Figure 1C, the TVB-N content of traditional air-dried meat samples from different regions of Ordos ranged from 0.09 g/kg to 0.15 g/kg. The EC group exhibited the highest TVB-N content, which is related to the stronger protein decomposition ability of the dominant microbial community in this region. These may affect the rate of protein degradation and TVB-N accumulation, leading to a relatively high TVB-N value in mature air-dried meat. This finding is consistent with the results reported by Chen [32]. The WC group exhibited the lowest TVB-N content. Results indicated significant differences in TVB-N content among samples from different regions (*p* < 0.05); however, all the values remained within the normal range and did not exceed the prescribed limit [33].

TBARs content is commonly used to assess the degree of lipid oxidation and rancidity in meat products. It is mainly used to evaluate the formation of secondary oxidation products such as malondialdehyde. A higher TBARs content indicates a more severe degree of lipid oxidation [34]. As shown in Figure 1D, the relative TBARs content in traditional air-dried meat samples from different regions of Ordos ranged from 0.00117 g/kg to 0.00142 g/kg, with significant differences observed among the groups (*p* < 0.05). The WC group exhibited the lowest relative TBARs content (0.00117 g/kg) among all the tested samples, whereas the DC group showed the highest. This phenomenon may be attributed to lipases secreted by spoilage bacteria such as *Pseudomonas*, which decompose fats to generate free fatty acids and thus promote lipid oxidation, resulting in elevated TBARs values. Additionally, an excessively long air-drying period may prolong the duration of lipid oxidation, leading to an exponential increase in TBARs values over time. This finding is generally consistent with the results reported by Najar [35]. In meat products, a TBARs value ranging from 0.0006 g/kg to 0.0028 g/kg is considered normal [36]. All the traditional air-dried meat samples from Ordos fell within this threshold, thereby ensuring the flavor and quality of the product.

### 3.2. Sensory Evaluation of Ordos Traditional Air-Dried Meat

To clarify the differences in sensory quality of samples from different producing regions, four core sensory indicators (color, aroma, tissue composition, and texture) were evaluated, and inter-group differences were tested via one-way analysis of variance (ANOVA). The results are presented in Table 2. The results showed that significant differences were observed in the total sensory scores and individual sensory attribute scores among the five sample groups (*p* < 0.05). The EC group (77.7 ± 1.10) had the highest total sensory score, which was significantly higher than that of the other groups (*p* < 0.05). This group exhibited excellent performance in color, texture and aroma, with the best overall sensory quality.

The YC and EHC groups showed balanced scores in all sensory attributes, and no significant difference was found in their total scores (*p* > 0.05). Both groups presented stable natural fermentation flavor and consistent product quality. The DC group (20.1 ± 0.88) had the highest score for tissue composition, which was significantly higher than that of the YC group (*p* < 0.05). However, its texture score (18.1 ± 1.05) was significantly lower than those of the EC and WC groups (*p* < 0.05), indicating insufficient textural firmness. The textural properties of the DC group could be improved by optimizing natural fermentation parameters and adjusting the air-drying process. The WC group had the lowest total sensory score, with generally low scores in all attributes, especially in color and tissue composition. Its overall sensory quality can be effectively enhanced by optimizing local natural fermentation conditions and improving the air-drying procedure.

### 3.3. Analysis of Microbial Community Composition in Traditional Air-Dried Meat from Ordos

#### 3.3.1. Alpha Diversity Analysis

**Figure 2:**
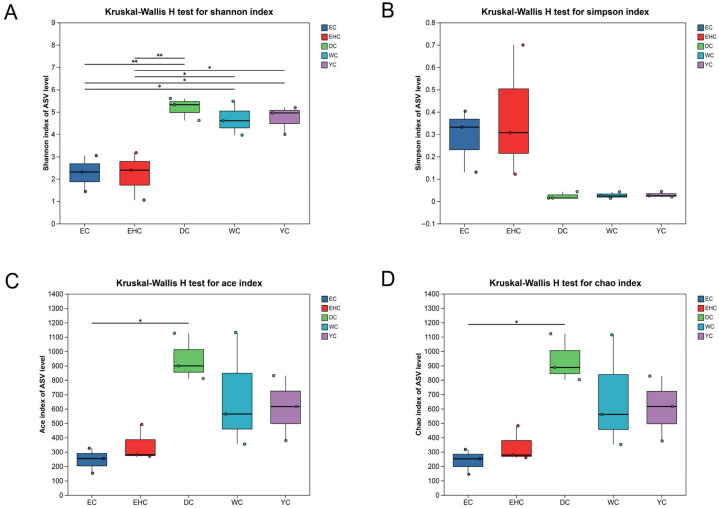
Differences in Bacterial Community composition Indices. (**A**) Shannon index of traditional air-dried meat samples from different regions of Ordos. (**B**) Simpson index of traditional air-dried meat samples from different regions of Ordos. (**C**) Ace index of traditional air-dried meat samples from different regions of Ordos. (**D**) Chao1 index of traditional air-dried meat samples from different regions of Ordos. Abbreviations: DC, Dalad Banner; EHC, Otog Banner; EC, Otog Front Banner; WC, Wushen Banner; YC, Ejin Horo Banner. * *p* < 0.05, significant difference; ** *p* < 0.01, extremely significant difference.

Alpha diversity was analyzed by Chao1, Ace, Shannon and Simpson indexes to reflect the richness and diversity of microbial communities. The Simpson and Shannon indices focus on quantitative characterization of biodiversity with consideration of species evenness. Specifically, a lower Simpson index value and a higher Shannon index value indicate greater microbial community diversity. In this study, alpha diversity analysis was carried out on traditional air-dried meat samples collected from different regions of Ordos. As shown in Figure 2A,B, the Shannon index of samples in the DC group was extremely significantly higher than that of the EHC and EC groups (*p* ≤ 0.01), while the Shannon index of the YC and WC groups was significantly higher than that of the EC and EHC groups (0.01 < *p* ≤ 0.05). No significant differences were observed in the Simpson index among samples from different regions (*p* > 0.05). The Simpson index of the EHC group was higher than that of the other groups, and the values of the DC, YC, and WC groups were almost evenly distributed. In summary, the WC group had the highest bacterial community diversity, while the EHC group had the lowest, and the diversity of other groups was between them. Chao1 and Ace indexes reflect the species richness of microbial communities, and higher values represent richer species. As shown in Figure 2C,D, the Chao1 index of samples in the DC group was significantly higher than that of the EC group (*p* ≤ 0.05); similarly, the Ace index of the DC group was significantly higher than that of the EC group (*p* ≤ 0.05). The Chao1 and Ace indices of the other groups were almost evenly distributed, with no significant differences observed (*p* > 0.05). These results indicate that the total number and richness of microbial communities in the DC group were greater than those in the other groups of samples.

#### 3.3.2. Beta Diversity Analysis

**Figure 3:**
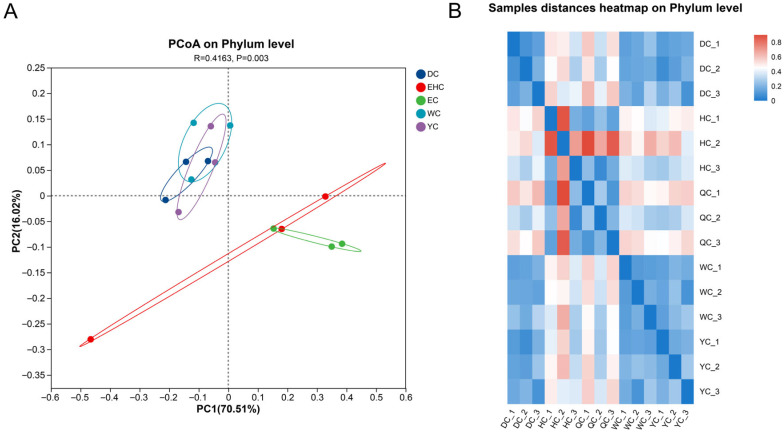
Beta Diversity Analysis. (**A**) PCoA Plot of Traditional Air-Dried Meat from Different Regions of Ordos. (**B**) Hierarchical Clustering Heatmap of Traditional Air-Dried Meat from Different Regions of Ordos. Abbreviations: DC, Dalad Banner; EHC, Otog Banner; EC, Otog Front Banner; WC, Wushen Banner; YC, Ejin Horo Banner.

All samples used mutton as raw material, but regional climate and processing techniques led to differences in microbial composition. Therefore, to investigate the composition and differences in bacterial communities in samples from different regions of Ordos, hierarchical clustering was performed, and the principal coordinate analysis (PCoA) plot of bacterial communities was constructed based on the sample distance matrix. This allowed for clear visualization of the clustering distances among samples. In the PCoA plot, shorter distance between samples indicated greater similarity in microbial community composition. As shown in Figure 3A, the relative abundance of bacteria in traditional air-dried meat samples from different regions of Ordos accounted for 70.51% of the variance in the first principal component (PC1) and 16.02% in the second principal component (PC2). The EHC and EC groups were far apart from the other groups in the plot, although an overlapping region was observed between the two groups. This indicated that the composition of bacterial communities in these two regions was different from that of the other regions, although the two groups also shared certain community composition similarities. In contrast, samples from the DC, WC, and YC groups were closely clustered and mostly distributed within the same region, suggesting no significant differences in composition of bacterial communities among them.

To further examine the relationship between the dominant microbial communities in Ordos samples and their distribution across sampling locations, a hierarchical clustering dendrogram was constructed. As shown in Figure 3B, samples were clustered into two main branches: EHC group formed an independent cluster, and the other four groups were clustered into another branch; EC group formed an independent sub-cluster, and DC, WC, YC groups were further clustered together. The clustering difference is caused by geographical environment and processing techniques, and the results of hierarchical clustering are consistent with PCoA, confirming the regional specificity of microbial communities.

#### 3.3.3. Analysis of Bacterial Community Composition in Traditional Air-Dried Meat from Ordos

**Figure 4:**
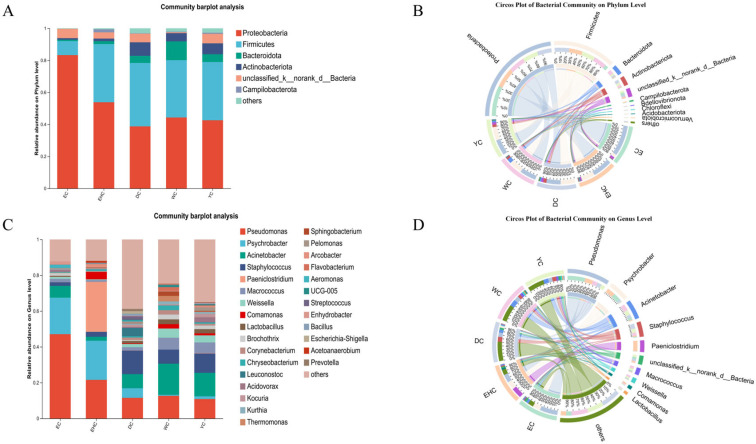
Analysis Chart of Microbial Community. (**A**) Analysis of Bacterial Community Composition at the Phylum Level; (**B**) Circos Plot of Bacterial Community at the Phylum Level; (**C**) Analysis of Bacterial Community Composition at the Genus Level; (**D**) Circos Plot of Bacterial Community at the Genus Level.

Based on the results of taxonomic analysis, the species abundance and dominant bacterial phyla at the phylum level were identified in traditional air-dried meat samples from different regions of Ordos. As shown in Figure 4A,B, more than 30 bacterial phyla were detected, with the top four phyla with the highest relative abundance being *Proteobacteria*, *Firmicutes*, *Bacteroidota*, and *Actinobacteriota*. *Proteobacteria* accounted for the highest relative abundance across all groups, with a maximum of 83.28% observed in the EC group. The relative abundances of *Firmicutes* in the DC, EHC, WC, and YC groups were relatively close, with the highest proportion (39.58%) observed in the DC group. The WC (11.72%) and YC (10.23%) groups contained a relatively high abundance of *Bacteroidota*. A high abundance of *Campylobacterota* was detected in the DC, WC, and YC groups, with significantly higher abundance compared to other groups. Previous studies have demonstrated that *Firmicutes* and *Proteobacteria* are the dominant phyla in traditional air-dried meat [37], which is consistent with the findings of this study.

The composition of bacterial communities at the genus level in samples from different regions of Ordos is presented in Figure 4C,D. The dominant bacterial genera included *Pseudomonas*, *Psychrobacter*, *Acinetobacter*, *Staphylococcus*, *Paeniclostridium*, *Weissella*, *Macrococcus*, and *Lactobacillus Pseudomonas* exhibited the highest relative abundance in the EC group, whereas its abundance was the lowest in the DC group. As a common bacterial group involved in natural fermentation, *Pseudomonas* may participate in the formation of flavor compounds through the decomposition of proteins and fats, but its excessive proliferation may pose spoilage risk [38]. Among the sample groups, *Paeniclostridium* showed the highest relative abundance in the EHC group and was identified as the core dominant genus in this group. *Staphylococcus* was the main dominant genus in the DC group. *Acinetobacter* accounted for the highest relative percentage in the WC and YC groups, serving as the primary dominant genus in these two groups. These results are consistent with the findings reported by Qin [39]. In summary, the dominant genera in Ordos air-dried meat have obvious regional differences, which are affected by environment, raw materials and processing techniques; these dominant genera can adapt to the low Aw environment of air-dried meat. This finding is consistent with the results reported by Sun [40].

### 3.4. Analysis of Volatile Flavor Compounds in Traditional Air-Dried Meat from Ordos

#### 3.4.1. Analysis of Volatile Flavor Compound Composition

Significance analysis of the main volatile flavor compounds in traditional air-dried meat samples from different regions of Ordos can provide a more intuitive assessment of the contribution of each sample group to the overall volatile flavor profile [41].

As shown in Appendix A (Table A1), a total of 91 volatile flavor compounds, including twenty-two alcohols, twenty-two alkanes, fourteen aldehydes, Seven ester flavor compounds, six ketones, four alkenes, five ethers, three aromatic hydrocarbons, three amines, two acids, and two pyrazines, were detected in the traditional air-dried meat samples from different regions of Ordos. Significant differences (*p* < 0.05) were observed in both the type and relative content of volatile aroma components with high contribution among samples from different regions.

Alcohols are mainly derived from the degradation of secondary hydroperoxides of fatty acids or the reduction of carbonyl compounds. As important synthetic precursors, they are extensively generated during lipid oxidation. The high activity of lipid oxidation and carbonyl reduction reactions further confirms their extensive production [42]. Compared with saturated alcohols, unsaturated alcohols play a more significant role in flavor formation and contribute more strongly to the overall flavor profile [43]. 1-nonen-3-ol, characterized by mushroom-like, lavender-like, and hay-like aromas, exhibited the highest relative content in the DC and YC groups. Trans-2-octen-1-ol, with fresh and grassy notes, showed the highest relative content in the EC, EHC, and WC groups. 4-Methyl-5-decanol, 2,4-decadien-1-ol, 2-pentadecyn-1-ol, and 1-dodecen-3-ol are unsaturated alcohols that impart mild floral, fruity, and grassy aromas to traditional air-dried meat; these compounds were also identified as alcohol-specific flavor compounds in the EHC group.

Hydrocarbons include flavor compounds such as alkanes, terpenes, alkenes, and aromatic hydrocarbons. A large variety of alkanes were detected in all the air-dried meat samples. Volatile alkane flavor compounds are mostly derived from the cleavage of alkoxy free radicals of fatty acids, and variations in alkane contents may be attributed to differences in fatty acid composition of the raw mutton [44]. 2,4-Dimethylhexane and 3,5-dimethyloctane were identified as alkane-specific flavor compounds in the EHC group, whereas 3,5,24-trimethyltetradecane was uniquely detected in the EC group. In addition to alkanes, terpenes constituted another major class of detected hydrocarbons, including 1-caryophyllene, dipentene, longifolene, valencene, and d-limonene. Terpenes are mostly derived from spices: 1-caryophyllene imparts mild clove and pepper-like aromas, whereas valencene and longifolene contribute citrus-like notes. Aromatic hydrocarbon flavor compounds included p-xylene, m-xylene, and ethylbenzene, whereas alkene flavor compounds comprised cyclooctatetraene and styrene. Hydrocarbons are not the main flavor contributors, but they provide a basic flavor background and synergize with aldehydes, ketones and alcohols to form the unique flavor of the product [45].

Aldehydes are recognized as important aroma components in meat products, generally produced during the oxidation of unsaturated fatty acids or the degradation of sugars. Nonanal, hexanal, and 3-methylhexanal constituted the aldehydes with relatively high contents in the traditional air-dried meat samples. Among these, nonanal exhibited the highest content, was detected in all samples from different regions of Ordos, and was also the most abundant aldehyde in each sample group. Characterized by rose-like and citrus-like aromas, nonanal better highlights the fatty aroma of traditional air-dried meat with high fat content. Its precursor is linoleic acid [46], and an elevated levels of this precursor contributes to the increased nonanal levels observed across all sample groups. Generally, aldehydes with lower sensory threshold values exert more significant effects on meat flavor. Therefore, despite their lower content compared with esters and alcohols, aldehydes play a more prominent role in enhancing the flavor of traditional air-dried meat [47]. When combined with ester flavor compounds, aldehydes contribute to the complex “fruity-fatty-green” flavor profile.

Seven ester flavor compounds were detected in the traditional air-dried meat samples from Ordos. Among all volatile compounds, esters exhibit fruity aromas and are mainly derived from the hydrolysis of short-chain fatty acids, serving as an important aroma component in traditional air-dried meat. The EC group showed the highest relative ester content, with ethyl lactate and ethyl 5-methylnonanoate identified as ester-specific flavor compounds in traditional air-dried meat. Vinyl hexanoate, characterized by apple-like and grassy aromas, was the most abundant ester compound common to all traditional air-dried meat samples from Ordos.

A total of six ketones, five ethers, three amines, two pyrazines, and two acids were detected in the traditional air-dried meat samples from Ordos. Ketone flavor compounds are mainly generated through three pathways: the Maillard reaction between glucose and amino acids, the oxidative degradation of fatty acids, and microbial metabolism, all of which produce sensory compounds with extremely low sensory threshold values. Although ketones contribute less to flavor than aldehydes and alcohols, they play an irreplaceable role in the flavor formation of traditional air-dried meat. Diketones are the initial products of the Maillard reaction, contributing meaty and buttery aromas to meat products; longer carbon chains are associated with more intense flavors. 2,5-Octanedione, with grassy and milky aromas, was the most abundant ketone in the EC and YC groups. 2-Ethylhexyl vinyl ether was identified as a volatile flavor compound unique to the EHC and EC groups, whereas n-octyl ether was specific to the YC group. N, N-Dibutylformamide and propyl neopentylamine exhibited the highest relative contents in the WC group. Propanamide acid, with the highest content in the WC group, imparts a sweet aroma to foods and may participate in the Maillard reaction to generate unique flavors.

Pyrazine compounds arise from the thermal decomposition of proteins and amino acids, as well as from the Maillard reaction between reducing sugars and amino acids, and contribute strongly to the color and flavor of traditional air-dried meat [48]. 2,6-Dimethylpyrazine and 2,5-dimethylpyrazine were identified as volatile flavor compounds specific to the EC group. Pyrazines have extremely low threshold values and mainly contribute roasted coffee-like and nutty aromas.

#### 3.4.2. Analysis of the Characteristic Flavors of Traditional Air-Dried Meat from Ordos Based on ROAVs

According to the ROAV evaluation system, volatile compounds with ROAV > 1 are generally recognized as making a dominant contribution to the overall aroma of food, whereas those with ROAV ranging from 0.1 to 1 serve an auxiliary flavor-enhancing compounds [49]. It is worth noting that GC-MS revealed that some compounds, despite being present in high concentrations, exhibited low ROAVs owing to their high olfactory thresholds and thereby contributed minimally to the characteristic aroma. Conversely, certain trace components, owing to their extremely low sensory thresholds, may achieve high ROAV values and consequently be key contributors to the characteristic aroma of the product [50].

Therefore, this study employed the ROAV method to identify and analyze the main characteristic volatile flavor compounds in traditional air-dried meat samples from Ordos. Relevant threshold data were retrieved from published studies [51,52]. As shown in Appendix A (Table A2), ROAVs were calculated for 37 volatile compounds, and a total of 11 flavor compounds (ROAV > 1) were screened as key contributors to the characteristic flavors of traditional air-dried meat. These included an alcohol compound (4-methyl-5-decanol), five aldehyde compounds (heptanal, hexanal, nonanal, 3-methylhexanal, and myristaldehyde), a ketone compound (2,5-octanedione), three ester compounds (ethyl octanoate, ethyl decanoate, and vinyl hexanoate), and a terpene compound (1-caryophyllene). Based on the quantitative evaluation criteria for flavor contribution of ROAV, compounds with an ROAV > 1 are defined as the core key components that determine the characteristic flavor of the sample, and the proportion of each component category directly reflects its contribution to the overall flavor. In this study, aldehydes accounted for 45.5% of the key flavor compounds with ROAV > 1, representing the highest proportion among all flavor categories. This directly verifies the dominant role of aldehydes in the flavor formation in traditional Ordos air-dried meat from a quantitative ROAV perspective. In addition, six volatile flavor compounds (0.1 ≤ ROAV ≤ 1), namely 1-octen-3-ol, decanal, isovaleraldehyde, ethyl octanoate, ethyl decanoate, and valencene played an important modifying role in the overall flavor profile of traditional air-dried meat from Ordos.

The number of key flavor compounds (ROAV > 1) in each region was: EHC (7) = EC (7) > WC (6) = YC (6) > DC (5). 4-methyl-5-decanol was unique to EHC, ethyl octanoate and ethyl decanoate were unique to EC; nonanal had the highest ROAV in all samples, confirming the dominant role of aldehydes. These key and auxiliary compounds together form the unique meaty and fatty flavor of Ordos air-dried meat.

### 3.5. Correlation Analysis of Microbial Community Composition in Traditional Air-Dried Meat from Ordos

#### 3.5.1. Correlation Between Microbial Community Composition and Flavor of Traditional Air-Dried Meat from Ordos

**Figure 5:**
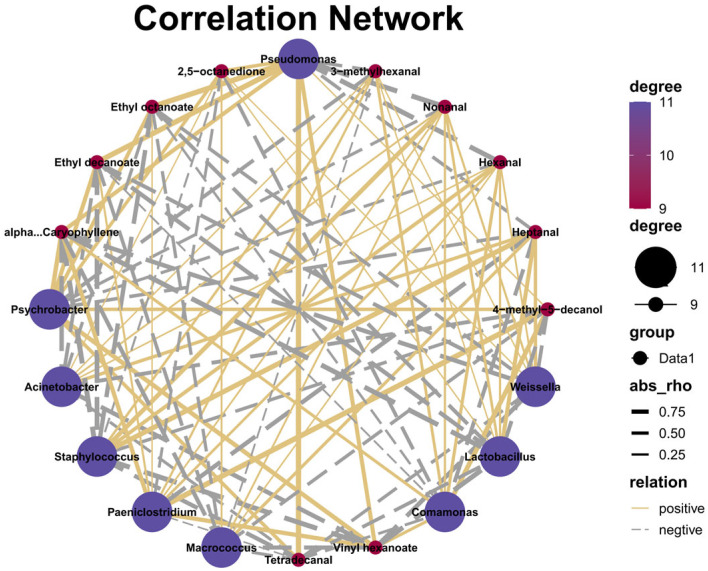
Correlation Analysis of Dominant Flora and Key Volatile Flavor Compounds in Traditional Air-Dried Meat from Different Regions of Ordos.

The volatile flavor compounds produced in traditional air-dried meat samples from different regions of Ordos are closely related to their microbial community compositions. To further clarify the relationship between microbial communities and flavor development, Pearson correlation analysis was performed combined with significance data screening (*p* < 0.05). The analysis identified correlations between dominant bacterial genera (with relative abundance > 1%) and characteristic volatile flavor compounds in traditional air-dried meat from different regions of Ordos. As shown in Figure 5, positive correlations are represented by solid yellow lines, negative correlations by dashed gray lines, with thickness of the lines indicating the strength of the correlation. *Pseudomonas* showed an extremely significant positive correlation (*p* < 0.01) with three key volatile flavor compounds, namely ethyl decanoate, myristaldehyde, and ethyl octanoate. In addition, it exhibited negative correlations with hexanal and nonanal. *Staphylococcus* was positively correlated with heptanal and showed significant negative correlations with 1-caryophyllene and vinyl hexanoate. Paeniclostridium exhibited an extremely significant positive correlation (*p* < 0.01) with 4-methyl-5-decanol.

In summary, different dominant genera have different regulatory effects on flavor compounds: positive correlation promotes the production of flavor substances, and negative correlation inhibits their formation.

#### 3.5.2. Correlation Analysis Between Microbial Community Composition and Quality of Traditional Air-Dried Meat from Ordos

The microbial community composition in traditional air-dried meat from Ordos is closely associated with food quality control indicators. Pearson correlation analysis was performed to determine the correlations between dominant bacterial genera (with relative abundance > 1%) and the contents of TBARs and TVB-N in traditional air-dried meat samples from different regions of Ordos. As shown in Table 3, *Pseudomonas* was negatively correlated with pH and TBARs, but positively with TVB-N; Psychrobacter exhibited the same correlations: negative with pH and TBARs, and positive with TVB-N. *Staphylococcus* was positively correlated with TBARs but negatively with TVB-N. The remaining genera were negatively correlated with both TBARs and TVB-N, but positively with pH. In summary, the microbial community composition of traditional Ordos air-dried meat was correlated with pH, TBARs, and TVB-N. *Pseudomonas* was significantly inhibited by pH, whereas *Weissella* and other genera showed a strong positive correlation with pH. This pattern may regulate the pH microenvironment of the meat via microbial metabolic balance, thereby affecting product quality and shelf-life stability. High abundance of *Pseudomonas* and *Psychrobacter* may promote food spoilage, whereas high *Staphylococcus* abundance may exacerbate lipid oxidation. The differential correlation is caused by the low Aw environment of air-dried meat, which regulates the secretion of protease and lipase by microorganisms, and affects product quality through microbial metabolism and ecological competition [40].

## 4. Conclusions

This study systematically investigated the physicochemical properties, microbial community structure, and volatile flavor profiles of traditional air-dried meat from different production regions of Ordos. The results showed that the physicochemical quality of the traditional air-dried meat was maintained at favorable levels. Aldehydes were identified as the dominant components responsible for the characteristic flavor of the product. The bacterial communities were overwhelmingly dominated by the phyla Proteobacteria and Firmicutes, with significant differences in community composition observed across different production regions. The core dominant genera mainly included *Pseudomonas*, *Psychrobacter*, and other related taxa, which were the key microbial groups driving product quality and flavor formation. Correlation analysis demonstrated that the dominant bacterial genera were significantly correlated with the formation of key flavor compounds, and jointly shaped the physicochemical quality and flavor characteristics of the product. These findings elucidate the core factors influencing the quality and flavor of traditional Ordos air-dried meat, and provide a theoretical basis for its standardized production and industrial development.

## Figures and Tables

**Table 1:** Sensory Evaluation Form.

Dimension	19–25 Points	13–18 Points	7–12 Points	1–6 Points
Color	Natural reddish-brown or light brownish-red, uniform and glossy, consistent with the inherent color characteristics of grassland air-dried meat	Reddish-brown, relatively uniform, with slight gloss	Overly dark or pale, uneven color distribution, dull luster	Dull or grayish, no gloss, with obvious color defects
Aroma	Rich in the unique aroma of grassland air-dried meat, with harmonious fat notes and subtle fermented undertones, free of gamey or rank odors	Characteristic grassland air-dried meat aroma, balanced fat notes, no off-odors	Faint meat aroma with slight fermented notes, no obvious off-odors	No detectable meat aroma, with gamey, rancid, or other off-odors
Tissue composition	Neat and dense cross-section, distinct muscle texture, no visible cracks	Relatively neat cross-section, clear texture, with only minor cracks	Uneven cross-section, blurred texture, with obvious cracks	Loose and fragmented cross-section, disordered texture, with severe cracks
Texture	Firm and chewy, moderate hardness, full mouthfeel, no dry or fibrous texture	Relatively firm, moderate chewiness, no significant looseness	Slightly loose, insufficient chewiness, soft texture	Lax and crumbly, no chewiness, easily broken

**Table 2:** Sensory Evaluation Results of Traditional Air-Dried Meat from Different Regions of Ordos. Note: Data are presented as mean ± standard deviation (SD) of the scores from 10 panelists. Different letters in the same row indicate significant differences among different sample groups (*p* < 0.05). Abbreviations: EC, Otog Front Banner; DC, Dalad Banner; YC, Ejin Horo Banner; WC, Wushen Banner; EHC, Otog Banner.

Evaluation Index	EC	DC	YC	WC	EHC
Color	21.6 ± 0.71 ^a^	20 ± 0.78 ^bc^	20.7 ± 0.74 ^ab^	19 ± 0.52 ^c^	20.2 ± 0.41 ^bc^
Aroma	16.3 ± 0.63 ^a^	14.6 ± 0.63 ^c^	15.3 ± 0.52 ^b^	14.6 ± 0.54 ^c^	15.7 ± 0.86 ^ab^
Tissue composition	19.2 ± 0.82 ^ab^	20.1 ± 0.88 ^a^	19.6 ± 0.80 ^ab^	18.5 ± 0.70 ^b^	18.8 ± 0.74 ^b^
Texture	20.6 ± 0.59 ^a^	18.1 ± 1.05 ^c^	19.3 ± 0.59 ^b^	20 ± 0.69 ^ab^	19 ± 0.77 ^bc^
Total Score	77.7 ± 1.10 ^a^	72.8 ± 1.15 ^c^	74.9 ± 1 ^b^	72.1 ± 1.05 ^c^	73.7 ± 1.08 ^bc^

**Table 3:** Table of Dominant Bacterial Genera and Quality Analysis of Traditional Air-Dried Meat from Different Regions in Ordos.

Name	*Pseudomonas*	*Psychrobacter*	*Staphylococcus*	*Acinetobacter*	*Weissella*	*Paeniclostridium*
pH	−	−	+	+	+	+
Rho	−0.6883	−0.1727	0.5457	0.2783	0.6452	0.4999
TBARs	−	−	+	−	−	−
Rho	−0.1543	−0.0181	0.6079	−0.3154	−0.3194	−0.2884
TVB-N	+	+	−	−	−	−
Rho	0.7256	0.7868	−0.5199	−0.3095	−0.3113	−0.0568

Note: “−” indicates negative correlation; “+” indicates positive correlation.

## Data Availability

The raw 16S rRNA gene sequencing data generated in this study have been deposited in the NCBI Sequence Read Archive (SRA) under the BioProject accession number PRJNA1432322 (Temporary Submission ID: SUB16040136), which is scheduled for public release on 7 March 2026 and accessible at: https://www.ncbi.nlm.nih.gov/sra/PRJNA1432322 (accessed on 22 April 2026). The remaining data supporting the findings of this study are available from the corresponding authors upon reasonable request.

## Abbreviations

The following abbreviations are used in this manuscript:

TVB-N	Total volatile basic nitrogen
TBARs	Thiobarbituric Acid Reactive Substances
ROAV	Relative odor activity value
A_w_	Water Activity
MgO	Magnesium Oxide
TCA	Trichloroacetic Acid
CCS	Circular Consensus Sequencing
SPME	Solid-Phase Microextraction
GC-MS	Gas Chromatography-Mass Spectrometry
PC	Principal component

## Appendix A

**Table A1:** Analysis Table of Relative Contents of Volatile Flavor Compounds in Traditional Air-Dried Meat from Different Regions of Ordos (%).

Name	DC	EHC	EC	WC	YC
1-Eicosanol	0.10 ± 0.03 b	-	-	0.53 ± 0.18 a	-
1-Nonen-3-ol	3.50 ± 0.45 b	3.99 ± 0.20 a	2.52 ± 0.24 c	2.33 ± 0.25 c	2.52 ± 0.18 c
1-Pentanol	0.43 ± 0.04 a	0.48 ± 0.04 a	0.18 ± 0.13 b	0.45 ± 0.07 a	-
1-Octanol	0.84 ± 0.13 b	1.45 ± 0.11 a	1.28 ± 0.09 a	-	0.93 ± 0.09 b
1-Octen-3-ol	3.50 ± 0.04 b	3.99 ± 0.20 a	2.51 ± 0.24 c	2.3 ± 0.25 c	2.52 ± 0.18 c
2-Propyl-1-heptanol	0.45 ± 0.15 a	-	-	0.22 ± 0.09 b	-
2-Propyl-1-pentanol	1.98 ± 0.19 a	1.58 ± 0.57 a	2.98 ± 1.62 a	1.71 ± 0.67 a	-
2-Butyl-1-octanol	0.26 ± 0.16 a	0.11 ± 0.03 a	0.22 ± 0.35 a	0.22 ± 0.19 a	0.23 ± 0.2 a
2-Ethylcyclobutanol	9.75 ± 1.80 a	8.72 ± 0.44 ab	5.23 ± 0.65 d	7.34 ± 0.53 c	-
2-Ethylhexanol	1.98 ± 0.19 ab	1.58 ± 0.57 ab	2.98 ± 1.62 a	1.71 ± 0.67 ab	1.29 ± 0.19 b
2-Vinyloxyethanol	0.40 ± 0.36 a	-	-	-	0.27 ± 0.01 b
1-Heptanol	0.29 ± 0.37 bc	0.60 ± 0.10 a	0.23 ± 0.02 c	-	0.34 ± 0.23 b
1-Nonadecanol	0.13 ± 0.01 a	0.12 ± 0.02 a	-	0.09 ± 0.02 b	-
Ethanol	1.41 ± 0.23 b	1.82 ± 0.68 b	2.23 ± 1.90 b	6.50 ± 1.09 a	0.84 ± 0.03 b
3-Methyl-1-butanol	0.43 ± 0.04 a	0.48 ± 0.03 a	0.18 ± 0.01 b	0.45 ± 0.66 a	-
3-Decyn-2-ol	-	0.08 ± 0.01 a	-	-	0.08 ± 0.17 a
1-Hexadecanol	-	0.24 ± 0.33 a	-	0.09 ± 0.01 b	0.99 ± 0.01 b
Cyclopentanol	-	0.67 ± 0.03 b	-	1.12 ± 0.12 a	-
4-Methyl-5-decanol	-	0.91 ± 0.96 a	-	-	-
2,4-Decadien-1-ol	-	0.41 ± 0.36 a	-	-	-
2-Pentadecyn-1-ol	-	0.14 ± 0.15 a	-	-	-
1-Dodecen-3-ol	-	0.11 ± 0.16 a	-	-	-
1,2-Epoxycyclooctane	0.39 ± 0.08 b	0.74 ± 0.06 a	-	0.42 ± 0.08 b	0.41 ± 0.02 b
2,6,10,14-Tetramethylheptane	0.25 ± 0.03 a	0.21 ± 0.01 b			0.27 ± 0.24 a
2-Methyldecane	0.25 ± 0.04 a	-	-	0.45 ± 0.07 b	-
3-Methyltridecane	0.11 ± 0.01 a	-	0.15 ± 0.06 a	0.11 ± 0.01 a	0.13 ± 0.01 a
3-Methylundecane	0.36 ± 0.03 a	-	-	0.28 ± 0.04 b	0.20 ± 0.17 a
4-Methyltetradecane	0.11 ± 0.01 a	-	-	-	-
9-Methylheptadecane	0.36 ± 0.03 a	-	-	-	0.34 ± 0.04 a
Decane	0.48 ± 0.04 a	0.15 ± 0.02 b	0.27 ± 0.28 ab	0.40 ± 0.06 a	0.52 ± 0.06 a
Dodecane	1.67 ± 0.17 a	0.73 ± 0.04 b	1.51 ± 0.98 ab	1.74 ± 0.27 a	1.67 ± 0.12 a
Nonadecane	0.21 ± 0.03 a	0.22 ± 0.03 a	0.13 ± 0.03 b	0.24 ± 0.05 a	0.28 ± 0.03 a
Hexadecane	0.71 ± 0.11 a	0.60 ± 0.07 ab	0.26 ± 0.08 bc	0.19 ± 0.06 c	0.46 ± 0.42 abc
Tetradecane	0.71 ± 0.11 a	0.65 ± 0.06 a	0.54 ± 0.23 a	0.68 ± 0.12 a	0.66 ± 0.24 a
Undecane	1.05 ± 0.08 a	0.40 ± 0.03 a	0.74 ± 0.81 a	1.04 ± 0.16 a	1.06 ± 0.12 a
n-Tridecane	1.05 ± 0.08 a	0.73 ± 0.04 a	1.31 ± 1.18 a	1.74 ± 0.27 a	1.47 ± 0.35 a
n-Octane	-	0.33 ± 0.03 b	-	0.65 ± 0.01 a	0.71 ± 0.07 a
1,2-Epoxyhexadecane	-	0.11 ± 0.02 a	0.10 ± 0.02 a	-	-
2,4-Dimethylhexane	-	0.33 ± 0.03 a	-	-	-
3,5-Dimethyloctane	-	0.15 ± 0.18 a	-	-	-
6-Methyloctadecane	-	-	0.07 ± 0.01 bc	0.08 ± 0.01 b	0.19 ± 0.09 a
3,5,24-Trimethyltetradecane	-	-	0.08 ± 0.02 a	-	-
2,6,10-Trimethyldodecane	-	-	-	0.43 ± 0.07 b	0.24 ± 0.05 c
2,6,10-Trimethyltetradecane	-	-	-	0.17 ± 0.06 a	0.07 ± 0.01 b
Benzaldehyde	0.98 ± 0.03 b	0.75 ± 0.03 b	3.63 ± 0.35 a	-	-
trans-2-Octenal	0.39 ± 0.08 b	0.74 ± 0.06 b	6.24 ± 0.47 a	0.42 ± 0.08 b	0.41 ± 0.02 b
trans-2-Decenal	0.13 ± 0.03 c	0.19 ± 0.03 b	0.57 ± 0.03 a	0.14 ± 0.03 bc	0.17 ± 0.01 bc
trans-2-Nonenal	0.54 ± 0.07 bc	0.74 ± 0.04 b	16.24 ± 0.70 a	0.70 ± 0.13 b	-
Heptanal	6.30 ± 0.25 a	4.63 ± 0.20 c	5.23 ± 0.65 bc	5.57 ± 0.71 ab	6.28 ± 0.26 a
Decanal	0.62 ± 0.04 a	0.55 ± 0.05 a	0.57 ± 0.03 a	0.44 ± 0.10 b	0.57 ± 0.03 a
Hexanal	9.75 ± 1.80 a	8.72 ± 0.44 ab	0.25 ± 0.02 d	7.34 ± 0.53 bc	6.88 ± 0.20 b
Nonanal	20.02 ± 0.72 a	14.19 ± 0.68 c	0.44 ± 0.01 d	12.61 ± 2.17 c	16.25 ± 0.73 b
Undecanal	0.11 ± 0.02 a	-	-	-	-
Pentanal	0.97 ± 0.19 a	-	0.37 ± 0.26 b	+	0.79 ± 0.02 a
3-Methylbutanal	0.32 ± 0.38 a	-	-	-	0.33 ± 0.43 a
n-Octanal	4.81 ± 0.26 b	6.22 ± 0.24 a	0.62 ± 0.02 d	3.47 ± 0.74 c	4.98 ± 0.35 b
3-Methylhexanal	6.30 ± 0.25 a	4.63 ± 0.20 c	0.10 ± 0.02 d	5.57 ± 0.71 b	-
Myristaldehyde	-	0.09 ± 0.01 b	0.49 ± 0.07 a	-	-
3,5-Octadien-2-one	0.32 ± 0.03 c	0.56 ± 0.01 a	0.18 ± 0.03 d	0.45 ± 0.08 b	-
Geranylacetone	0.14 ± 0.02 b	-	0.18 ± 0.04 ab	-	0.19 ± 0.03 a
2-Methyl-3-pentanone	-	0.67 ± 0.03 a	-	-	-
2-Heptanone	-	0.21 ± 0.01 a	0.12 ± 0.01 b	-	-
3-Undecanone	-	-	0.24 ± 0.02 a	-	-
2,5-Octanedione	-	-	2.46 ± 0.35 a	-	2.25 ± 0.17 a
p-Xylene	1.50 ± 0.97 b	2.65 ± 0.57 a	0.16 ± 0.01 c	3.43 ± 0.41 a	2.60 ± 0.18 a
m-Xylene	2.06 ± 0.01 b	2.65 ± 0.57 b	0.12 ± 0.01 c	3.43 ± 0.41 a	2.60 ± 0.18 b
Ethylbenzene	0.42 ± 0.03 b	0.38 ± 0.11 b	0.16 ± 0.01 c	0.70 ± 0.06 a	0.47 ± 0.05 b
2-Butoxyethanol	0.43 ± 0.04 a	-	-	-	0.21 ± 0.03 b
Decylether	0.08 ± 0.03 a	-	-	-	0.08 ± 0.01 a
Octadecyl vinyl ether	0.08 ± 0.01 a	-	-	-	0.10 ± 0.02 a
2-Ethylhexyl vinyl ether	-	0.23 ± 0.06 a	0.24 ± 0.02 a	-	-
n-Octyl ether	-	-	-	-	0.18 ± 0.01 a
1-Caryophyllene	0.15 ± 0.03 b	-	-	0.16 ± 0.02 b	0.56 ± 0.28 a
Limonene	0.27 ± 0.01 a	-	-	-	-
Longifolene	-	0.32 ± 0.07 a	-	-	-
Valencene	-	-	-	-	0.10 ± 0.01 a
O-Decylhydroxylamine	0.8 ± 0.01 a	-	0.11 ± 0.08 a	-	-
N,N-Dibutylformamide	-	-	-	0.23 ± 0.06 a	-
Propyl neopentylamine	-	-	-	0.23 ± 0.06 a	-
Vinyl hexanoate	2.36 ± 0.02 b	4.65 ± 0.14 a	4.16 ± 0.52 a	2.46 ± 0.35 b	2.25 ± 0.17 b
Ethyl octanoate	-	0.07 ± 0.06 b	0.25 ± 0.02 a		-
Ethyl nonanoate	-	0.13 ± 0.01 a	0.08 ± 0.01 b	-	-
Heptyl formate	-	0.60 ± 0.10 a	0.23 ± 0.02 b	-	-
Ethyl decanoate	-	0.13 ± 0.01 b	0.30 ± 0.03 a	-	-
Ethyl lactate	-	-	0.23 ± 0.06 a	-	-
Ethyl 5-methylnonanoate	-	-	0.18 ± 0.03 a	-	-
2,6-Dimethylpyrazine	-	-	0.15 ± 0.02 a	-	-
2,5-Dimethylpyrazine	-	-	0.15 ± 0.02 a	-	-
Propionamic acid	-	-	-	0.11 ± 0.03 a	-

Note: Different lowercase letters within the same row indicate significant differences (*p* < 0.05) among samples from different regions, as determined by Duncan’s multiple range test. Values with the same letter within the same row are not significantly different (*p* ≥ 0.05). “-” indicates the volatile compound was not detected in the sample.

**Table A2:** Table of ROAVs and Aroma Types of Traditional Air-Dried Meat from Different Regions in Ordos.

Name	Sensory Threshold	Relative Odor Activity Value					Aroma Type
		DC	EHC	EC	WC	YC	
1-Pentanol	4000	0.00	0.00	0.00	0.00	0.00	Fruity aroma
1-Octanol	110	0.00	0.01	0.01	0.00	0.01	Waxy aroma, Floral aroma, Fruity aroma
1-Octen-3-ol	10	0.23	0.37	0.20	0.24	0.20	Mushroom aroma, Lavender aroma, Hay-like aroma
2-Propyl-1-pentanol	4000	0.00	0.00	0.00	0.00	0.00	Herbal aroma, Green aroma
2-Butyl-1-octanol	110	0.00	0.00	0.00	0.00	0.00	Fruity aroma
2-Ethylhexanol	89	0.01	0.02	0.03	0.02	0.01	Citrus aroma, Sweet aroma
1-Heptanol	330	0.00	0.00	0.00	0.00	0.00	Herbal aroma, Fruity aroma
1-Hexanol	100	0.01	0.02	0.02	0.07	0.01	Fruity aroma, Floral aroma, Grass-like aroma
Isoamyl alcohol	250	0.00	0.00	0.00	0.00	0.00	Fruity aroma
4-Methyl-5-decanol	0.01	0.00	83.46	0.00	0.00	0.00	Floral aroma, Fruity aroma
2-Hexyl-1-octanol	270	0.00	0.00	0.00	0.00	0.00	Floral aroma, Fruity aroma, Fresh aroma
2,3-Butanediol	0.2	0.00	0.00	0.00	0.00	0.00	Buttery aroma
2,4-Dimethylcyclohexanol	10	0.00	0.00	0.00	0.00	0.00	Minty aroma, Floral aroma, Fruity aroma
Hexadecane	13,000	0.00	0.00	0.00	0.00	0.00	Waxy aroma
Benzaldehyde	3500	0.00	0.00	0.00	0.00	0.00	Sweet aroma, Almond-like aroma
Heptanal	3	1.36	1.41	1.40	1.91	1.68	Roasted aroma, Nutty aroma, Fresh aroma
Decanal	2	0.20	0.25	0.23	0.23	0.23	Citrus aroma, Floral aroma
Hexanal	4.5	1.41	1.77	0.04	1.68	1.22	Fruity aroma, Grass-like aroma
Nonanal	0.13	100.00	100.00	100.00	100.00	100.00	Rose-like aroma, Citrus aroma
Undecanal	5	0.01	0.00	0.00	0.00	0.00	Citrus aroma, Rose ester aroma
Isovaleraldehyde	0.4	0.52	0.00	0.00	0.00	0.65	Fruity aroma, Grass-like aroma
3-Methylhexanal	0.7	5.84	6.06	0.11	8.20	0.00	Nutty aroma
Myristaldehyde	0.053	0.00	1.54	7.34	0.00	0.00	Citrus aroma, Floral aroma
3,5-Octadien-2-one	70	0.00	0.01	0.00	0.01	0.00	Mushroom aroma, Floral aroma
2,5-Octanedione	0.54	0.00	0.00	3.65	0.00	3.33	Grass-like aroma, Milky aroma
Geranylacetone	60	0.00	0.00	0.00	0.00	0.00	Green aroma, Fruity and floral aroma, Waxy aroma
2-Methyl-3-pentanone	20	0.00	0.03	0.00	0.00	0.00	Fruity aroma, Ethereal aroma
2-Heptanone	600	0.00	0.00	0.00	0.00	0.00	Fruity aroma, Herbal aroma
3-Undecanone	7	0.00	0.00	0.03	0.00	0.00	Waxy aroma
3-Hydroxy-2-butanone	1000	0.00	0.00	0.00	0.00	0.00	Creamy aroma
4-Hydroxy-2-butanone	5000	0.00	0.00	0.00	0.00	0.00	Creamy aroma, Sweet aroma
Styrene	730	0.00	0.00	0.00	0.00	0.00	Sweet aroma, Floral aroma
Ethyl octanoate	0.2	0.00	0.31	1.00	0.00	0.00	Creamy aroma, Fruity aroma
Ethyl nonanoate	850	0.00	0.00	0.00	0.00	0.00	Fruity aroma
Ethyl decanoate	0.2	0.00	0.61	1.19	0.00	0.00	Sweet aroma, Creamy aroma
Ethyl lactate	150	0.00	0.00	0.00	0.00	0.00	Cheese-like aroma Milky aroma
Ethyl butyrate	250	0.00	0.00	0.00	0.00	0.00	Fruity aroma
Butyl lactate	140	0.00	0.00	0.00	0.00	0.00	Creamy aroma
Ethyl acetate	0.05	0.00	0.00	0.00	0.00	0.00	Ethereal aroma
Ethyl isovalerate	150	0.00	0.00	0.00	0.00	0.00	Fruity aroma
Ethyl isobutyrate	250	0.00	0.00	0.00	0.00	0.00	Fruity aroma
Ethyl valerate	2.5	0.00	0.00	0.00	0.00	0.00	Fruity aroma
Vinyl hexanoate	0.05	30.69	86.66	66.54	50.82	35.93	Green aroma, Fruity aroma
2,6-Dimethylpyrazine	54	0.00	0.00	0.00	0.00	0.00	Roasted aroma, Nutty aroma
2,5-Dimethylpyrazine	35	0.00	0.00	0.00	0.00	0.00	Roasted aroma, Nutty aroma
2,3,5,6-Tetramethylpyrazine	0.05	0.00	0.00	0.00	0.00	0.00	Nutty aroma, Coffee-like aroma, Roasted aroma
3-Methyloxirane-2-carboxylic acid	20	0.00	0.00	0.00	0.00	0.01	Grass-like aroma, Fruity aroma
1-Caryophyllene	0.1	0.99	0.00	0.00	1.68	4.49	Clove-like aroma, Peppery aroma
Valencene	0.13	0.00	0.00	0.00	0.00	0.64	Citrus aroma

Note: Threshold values were retrieved from the literature.
